# Thawing out frozen metabolic accidents

**DOI:** 10.1186/s12915-018-0621-5

**Published:** 2019-01-30

**Authors:** Dario Leister

**Affiliations:** Faculty of Biology, Ludwig-Maximilians-University Munich, Großhaderner Str. 2, 82152 Planegg-Martinsried, Germany

## Abstract

Photosynthesis and nitrogen fixation became evolutionarily immutable as “frozen metabolic accidents” because multiple interactions between the proteins and protein complexes involved led to their co-evolution in modules. This has impeded their adaptation to an oxidizing atmosphere, and reconfiguration now requires modification or replacement of whole modules, using either natural modules from exotic species or non-natural proteins with similar interaction potential. Ultimately, the relevant complexes might be reconstructed (almost) from scratch, starting either from appropriate precursor processes or by designing alternative pathways. These approaches will require advances in synthetic biology, laboratory evolution, and a better understanding of module functions.

## Frozen accidents: evolution of the genetic code

A key challenge in genetic engineering and synthetic biology is to *change the unchangeable*—i.e., to thaw the so-called frozen metabolic accidents (FMAs). The term FMA refers to processes that are thought to be immutable, because their modification requires altering multiple intertwined components at the same time. Historically, the term “frozen accident” was coined to explain certain characteristics of the standard genetic code (SGC), which is outlined in the following. In fact, the assignment of the 20 canonical amino acids to the 64 codons of the SGC is clearly non-random, i.e., related amino acids typically occupy contiguous areas in the codon table [1]. Several possible reasons for this have been proposed. Chemical interactions between amino acids and the tertiary structures of RNA-binding sites of codons (or anti-codons; “stereochemical theory”) [2], co-evolution of amino-acid biosynthesis and code structure (“coevolution theory”) [3], and selection for robustness (“error minimization theory”) [4] could all have contributed to the evolution of the SGC. Furthermore, the “frozen accident” perspective introduced by Francis Crick 50 years ago [5] explains the universality of the SGC by its effective fixation in the earliest life forms, such that any major change would be strongly selected against because it would immediately affect large numbers of proteins. This scenario does not require that the original assignment of codons occurred entirely by chance. In fact, the SGC displays clear signs of optimization: it is very robust, albeit not the most robust possible [6, 7], and the 20 canonical amino acids are thought to be virtually ideal for building soluble protein structures with close-packed cores [8]. But the frozen accident perspective also emphasizes that once the SGC assignment had been made, it became essentially immutable.

**Fig. 1.**
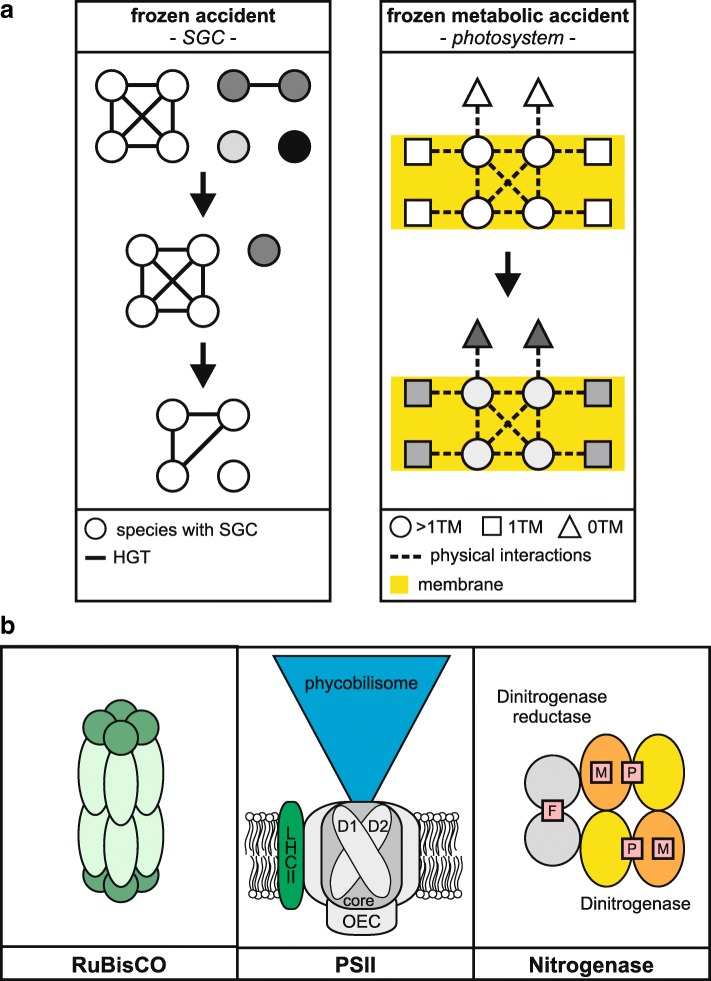
Evolution and instances of “frozen (metabolic) accidents.” **a** The standard genetic code (SGC) is a frozen genetic accident and became universal during early prokaryote evolution because of the eminent role of horizontal gene transfer (HGT) (*left side*). Each *circle* represents a species and different genetic codes are indicated by different fillings. Species with rare codes became extinct because they were less amenable to stable HGT. The code that remained became the SGC. Once it was universally adopted, it persisted even when HGT became less important. On the *right*, the evolution of photosystem subunits is schematically shown as an example of a FMA. In the thylakoid membrane (*yellow*), the core photosystem subunits (*circles*), having more than one transmembrane domain (TM), multiply interact with each other (*dotted lines*) and with their co-factors. They also interact to a lesser extent with other transmembrane photosystem proteins (with one TM, *rectangles*), as well as with peripheral photosystem proteins and soluble proteins (*triangles*). This leads to different rates and modes of evolution and sequence diversification (highlighted in *gray*, with increased variation being indicated by darker shading). The sequences of photosystem core subunits changed least during evolution and represent FMAs. **b** RuBisCO, PSII, and the molybdenum (Mo)-dependent cyanobacterial nitrogenase. RuBisCO consists of eight large (*light green*) and eight small (*dark green*) subunits and catalyzes carboxylation of ribulose-1,5-bisphosphate during the Calvin cycle. PSII contains a core with several subunits, of which D1 and D2 are shown. Other PSII subunits, including the oxygen-evolving complex (OEC), surround the core. In cyanobacteria, phycobilisomes act as antenna complexes, whereas light-harvesting complex II (LHCII) serves this function in eukaryotes. The Mo-nitrogenase is a two-component system comprising a heterotetrameric dinitrogenase complex with a P-cluster (P; Fe_8_S_7_) and an Fe-Mo cofactor (M), and a homodimeric dinitrogenase reductase with a Fe_4_S_4_ cluster (F)

Among the deleterious effects of codon reassignment, it is thought that inhibition of horizontal gene transfer (HGT) might have played a major role [1]. In fact, HGT remains a key factor in microbial evolution [9] and, assuming that the capacity to undergo HGT was essential for the early prokaryotes [10], even small alterations of the code would have had detrimental effects on HGT, genetically isolating the species affected and dooming them to extinction. HGT itself also explains the universality of the code. Advanced simulations of evolution starting with several genetic codes and extensive HGT lead to a single universal code [11–13] (Fig. 1a).

## The “frozen metabolic accident” concept

The term FMA was introduced by Paul Falkowski and coworkers to explain the evolutionary dynamics of photosynthetic genes [14]. Maximal co-evolution among photosynthetic genes occurs when their protein products physically interact with each other, and in prokaryotes, such genes are often clustered at the genomic level [14]. Thus, co-evolved photosynthetic proteins are found in thylakoid multiprotein complexes (photosystems I (PSI) and II (PSII), cytochrome *b*_6_*f* complex, and ATPase) and in soluble enzyme complexes like the tetrapyrrole biosynthesis enzyme magnesium protoporphyrin IX chelatase and the Calvin cycle enzyme ribulose-1,5-bisphosphate carboxylase/oxygenase (RuBisCO) [14] (Fig. 1b). The rate of amino acid substitution for photosynthetic proteins in the cores of the photosynthetic multiprotein complexes (with multiple transmembrane domains (TMs)) are indeed markedly lower than those of the small subunits surrounding the cores (with 1 TM), or peripheral or soluble photosynthetic proteins that lack TMs (Fig. 1a). This implies that interactions of proteins with other proteins, lipids, or cofactors constrain their evolution in the core photosynthetic apparatus of cyanobacteria and photosynthetic eukaryotes [14]. Interestingly, proteins involved in different multiprotein complexes also co-evolved, e.g., core proteins of PSI and PSII, as well as cytochrome *b*_6_*f* complex and NADH dehydrogenase proteins, suggesting that their functional linkage in thylakoid electron transport was responsible for their co-adaptation. Moreover, the stoichiometry of the output of the light reactions in terms of ATP and NADPH almost perfectly matches the needs of the Calvin cycle, indicating substantial co-evolution of entire functionally linked processes.

When environmental conditions changed several hundred million years ago, the functions of some of these conserved core components of photosynthesis were compromised. This holds in particular for their performance in the presence of oxygen and high light intensities—conditions that directly (oxygen) or indirectly (the ozone layer that enabled colonization of terrestrial habitats by blocking UV radiation) resulted from oxygenic photosynthesis. The two most prominently affected proteins are the D1 protein of PSII and the RuBisCO enzyme (Fig. 1b). Reactive oxygen species (ROS) directly damage D1 under high light intensities and inhibit its continuous replacement by newly synthesized copies [15–19], while RuBisCO’s propensity to employ oxygen instead of CO_2_ as substrate essentially wastes light energy [20]. Falkowski and coworkers estimated that these features of PSII and RuBisCO together reduce the potential overall efficiency of photosynthesis by at least 50% [14].

Like D1 and RuBisCO, nitrogenase (Fig. 1b)—the enzyme complex that fixes atmospheric nitrogen in prokaryotes—evolved before oxygen was freely available in the atmosphere, and the core proteins of cyanobacterial nitrogenases remained virtually unchanged following the transition to an oxidizing atmosphere [21, 22]. Hence, nitrogenase represents a third instance of a FMA [23]. Because of the oxygen sensitivity of the iron-sulfur clusters in these nitrogenases (Fig. 1b), an estimated 20 to 30% of marine nitrogenase activity is inhibited by O_2_. It is tempting to speculate that this evolutionary inflexibility might also explain why cyanobacterial nitrogenases were not endosymbiotically acquired by eukaryotes.

## Redressing the effects of FMAs

The functional shortcomings of RuBisCO, nitrogenase, and the D1 subunit of PSII have stimulated attempts to enhance their efficiency, aiming to improve crop yield and biomass production. These efforts have encountered many obstacles, which revealed additional facets of the consequences of FMAs. Exploiting interspecific diversity in RuBisCO activity, improving the activity of its auxiliary protein RuBisCO activase, and altering the levels of regulatory metabolites have been identified as promising ways to enhance the enzyme’s function in crop plants [20]. While these approaches are still at an early stage and are complicated by the fact that in crop plants the small and large subunits of the enzyme are encoded in the nucleus and chloroplast, respectively, they have already uncovered additional constraints on the evolution of RuBisCO. For instance, the enzyme’s subunits (Fig. 1b) co-evolve not only with each other [14], but also with its assembly factors [24]. This highlights why the modification of FMAs will often require the exchange of entire modules of co-evolved components, including structural proteins, auxiliary factors, and the genetic elements necessary for their efficient expression [25]. The number of auxiliary proteins implicated can even exceed the number of structural proteins present in the mature complex, in the case of RuBisCO prompting the coining of the term “rubiscosome,” describing its evolution from a stand-alone enzyme into an enzyme complex that involves various auxiliary factors [26, 27]. Indeed, it has only recently become possible to express a functional plant RuBisCO in *Escherichia coli* by co-expressing the large and small subunits of RuBisCo together with its five assembly factors [28]. This heterologous plant RuBisCO expression system promises to provide a way to test variants of the enzyme for enhanced function in the genetic workhorse *E. coli*. In fact, a non-native Calvin cycle or parts of it have previously been functionally reconstituted in *E. coli* [29, 30], yeast [31], or *Rhodobacter capsulatus* [32], allowing its optimization by directed evolution [33] or laboratory evolution [30, 34, 35]. Because several natural and synthetic autotrophic pathways besides the Calvin cycle exist, it is possible to design alternative carbon fixation cycles in plants, and such efforts are now underway [36–41].

With regard to nitrogenases, three strategies are available for enhancing biological nitrogen fixation in crops: (i) boosting the process in naturally plant-associated bacteria, (ii) inducing formation of the root nodules that permit symbiosis between crop plants and N_2_-fixing bacteria, and (iii) directly transferring prokaryotic nitrogenase genes into plant genomes [42, 43]. An important initial step was taken more than 40 years ago, when recombinant *E. coli* strains with nitrogenase activity were constructed by genetic engineering [44]. However, these transgenic strains exhibited much lower nitrogenase activity than the original host and could not support diazotrophic growth on nitrogen-free medium. *E. coli*-based systems were also used more recently to combine the nitrogenase with electron transport components from plant organelles as power supplies for future engineering of diazotrophy in cereal crops [45]. However, overcoming the FMA of nitrogenases by mitigating their oxygen sensitivity has not yet been accomplished.

**Fig. 2.**
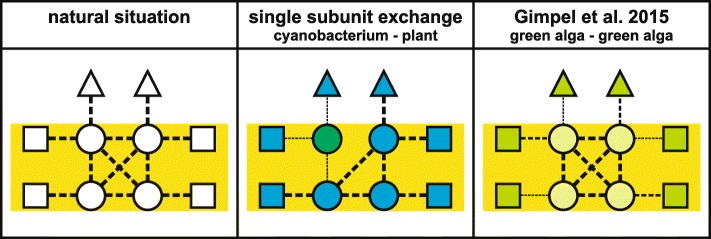
Current approaches to modifying the PSII core. *Left panel*: In PSII from cyanobacteria, algae and plants, the core subunits (*circles*) multiply interact (*dotted lines*) with each other and to a lesser extent with other transmembrane (*rectangles*) and peripheral (*triangles*) proteins. *Middle panel*: If core subunits in cyanobacteria (*blue*) are replaced by their plant pendants (*green*), their sequence differences may weaken or disrupt these interactions (*thinner dotted lines*), severely affecting the function of PSII. *Right panel*: Stephen Mayfield and co-workers [50] replaced an entire green algal PSII core with variants from other green algal species (indicated by a lighter shade of green). This would be expected to maintain the interactions between PSII core subunits, but might have affected interactions with other PSII proteins

While recombinant *E. coli* strains are available for RuBisCO and nitrogenase that provide a platform for modifying these suboptimal enzymes by genetic engineering and laboratory evolution, the situation for the PSII core proteins, including the D1 protein, is less favorable. Reconstitution of the photosynthetic light reactions in *E. coli* has proved to be a challenging task and has not yet been achieved. In fact, owing to their slow tempo of evolution (as expected for products of a FMA), the PSII core proteins are largely conserved between cyanobacteria and plants. Nevertheless, attempts to replace the cyanobacterial PSII core proteins D1, CP43, CP47, and PsbH with their counterparts from flowering plants (with which they share between 78 and 86% identity at the amino acid level) have been only partially successful [25] (Fig. 2). The resulting strains were either non-photoautotrophic or severely impaired in photosynthesis [46–49], demonstrating that the exchange of one component of this module of co-adapted PSII core proteins can markedly perturb the multiple interactions and the functionality of the entire PSII core.

In light of this limitation, Stephen Mayfield and co-workers proposed to replace the entire PSII core with its counterpart from another species, thereby maintaining the multiple intrinsic interactions within the PSII core that have evolved over millions of years in each photosynthetic species [50] (Fig. 2). To test this hypothesis, the six original core PSII genes (*psbA*, *B*, *C*, *D*, *E*, and *F*) from the chloroplast genome of the green alga *Chlamydomonas reinhardtii* were deleted and replaced by a single synthetic construct that contained the orthologous genes from *Volvox carteri* or *Scenedesmus obliquus* (two other green algal species) or, as a control, from *C. reinhardtii* [50]. In addition, the effect of replacing only subsets of the six genes was investigated. These experiments showed that (i) the strains reconstituted with the *C. reinhardtii* PSII gene sets showed the best photosynthetic performance, albeit lower than in the starting WT strain, and (ii) in the strains with replacements from *V. carteri* and *S. obliquus*, photosynthetic performance declined with increasing numbers of exchanged genes. These results imply that both the organization of the substitute genes in a synthetic construct (that might lack some *cis*-acting elements and their original operon structures, leading to suboptimal gene expression) and off-target effects of the PSII gene deletions (that might affect other operons and adjacent tRNA genes) in the *C. reinhardtii* chloroplast could decrease the photosynthetic performance of the transgenic strains. Moreover, the experiment could not clarify whether the roadblocks presented by FMAs can be truly removed by exchanging the entire ensemble (or module) of interacting and co-evolved proteins. In fact, the substituted green algal proteins were so similar with respect to their sequence—with average identities of between 93% (*S. obliquus*) and 98% (*V. carteri*) relative to the deleted original *C. reinhardtii* genes—that the strong negative effect on PSII function resulting from perturbations in gene expression in the host system used might have masked any subtle positive effect produced by the retention of the intrinsic interactions between the six co-adapted proteins. In consequence, a more appropriate experimental approach would be to replace entire cyanobacterial photosystem cores by their functional equivalents from higher plants [25], for which single subunit exchanges have proven to be clearly detrimental (see above). In such experiments, the exchange of multiple subunits might alleviate the strong negative effects of single subunit substitution, given that the experimental setup can achieve efficient expression of the introduced genes and efficient assembly of the corresponding proteins—the latter one might even require introduction of plant-specific assembly factors. Moreover, it might be necessary to replace not only the six core subunits, but also additional PSII proteins that physically and/or functionally interact with the PSII core.

## Novel approaches to bypassing roadblocks caused by FMAs

Functional resolution of FMAs by replacing the evolutionarily immutable protein (complexes) with more efficient variants is an enormous challenge. But the issue is not only of interest for evolutionary biologists, but would have significant implications for plant productivity, as it might be the only way to enhance photosynthetic performance and nitrogen fixation rates. With respect to photosynthesis, this endeavor involves the realization of an evolutionary development that never actually took place. One needs to create a primordial version of photosynthesis under aerobic conditions at high light intensities—the very conditions produced by the advent of oxygenic photosynthesis (see above)—and let it evolve. The outcome of this evolution might be to reduce the light sensitivity of the PSII reaction center and the current susceptibility of the carbon fixation cycle to the level of oxygen in the atmosphere.

How can this challenge be tackled? A “conservative” solution to create novel proteins would be to fully exploit the potential of the 20 canonical amino acids encoded by the SGC. Indeed, concepts for systematically designing entire libraries of “non-natural proteins” (based on the canonical amino acid repertoire) that can be employed in vivo to replace natural proteins have been presented, and proof of their practicability has been provided [51–53]. A second possibility is to expand or alter the genetic code. In fact, some organisms have succeeded in co-opting the two non-canonical amino acids selenocysteine and pyrrolysine into the code [1], and nonstandard amino acids with residues that have unusual chemical properties could be employed for the design of novel protein functions hitherto not possible with canonical amino acids. Thus, concepts for recoding genomes by re-assigning or deleting codons have been developed with the aim of enabling multivirus resistance, enhanced incorporation of nonstandard amino acids, or biocontainment by synthetic auxotrophy [54]. Such strains have been designed, generated, and subsequently streamlined by adaptive evolution [55–57].

**Fig. 3.**
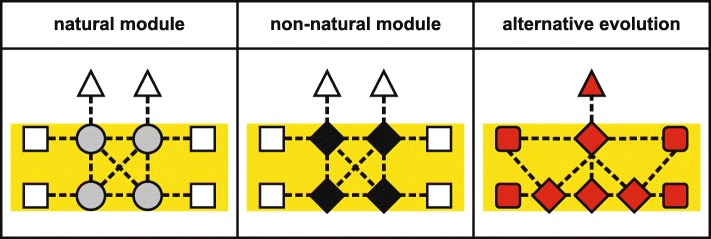
Overview of the three general strategies for the resolution of FMAs. *Left panel*: The module responsible for the FMA is replaced by a natural variant of the module. This might be effective if the alternative module has evolved beneficial characteristics due to high selective pressure. Possible trade-offs and interactions with proteins extraneous to the transferred module might need to be considered, but could be mitigated by laboratory evolution (see Fig. 4 for more details). *Middle panel*: The module is replaced by non-natural proteins that have the same interaction potentials but different sequences. This will very likely require a step-wise strategy, with successive replacement of individual proteins via a complementation-based approach, combined with streamlining of protein functions by laboratory evolution (see Fig. 5 for more details). *Right panel*: Alternative evolution of a primordial version of the FMA or its entire de novo design might be the only solution if the general design of the FMA is fundamentally flawed and cannot be corrected by the first two approaches (see Fig. 6 for more details)

These two concepts for the design of new proteins to counteract the repercussions of FMAs may be applied either “conservatively,” i.e., exploiting the opportunities offered by such scenarios (including the possibility of using non-natural proteins), or “unconventionally,” i.e., employing entirely new components and interactions. In addition, the recognition that all interacting/co-evolving components in a module may need to be altered must be taken into account. In consequence, three approaches for redressing the aftereffects of FMAs can be envisioned: exploiting (i) natural or (ii) non-natural variants of the components of the module concerned, and (iii) re-designing the corresponding biological process (almost) from scratch (Fig. 3). All three approaches can be expected to require extensive laboratory evolution [58] to streamline the novel processes, i.e., exploiting the high rate of evolution in microbial systems to optimize the expression, interactions, and performance of the introduced proteins for maximal implementation of the desired function. The third approach must follow an “alternative evolutionary trajectory,” either beginning with a primordial progenitor version of the process or re-designing the process from scratch. These three general concepts will now be discussed in the context of the re-engineering of the PSII core in order to bypass a FMA. In this instance, the difficulties arise from the special biophysical properties and pigment-binding sites of its components, as well as their multiple interactions and complex biogenesis [59–62].

## Exploiting natural sequence variations to enhance PSII functions

**Fig. 4.**
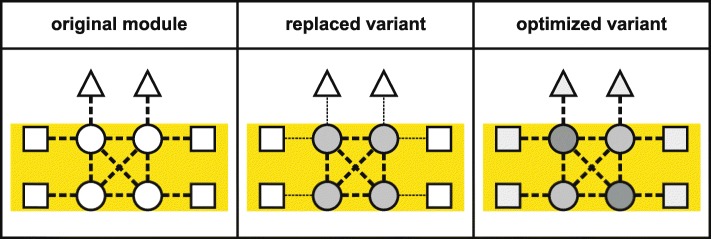
Replacing modules resulting from FMAs by natural variants. The original variant (*left*) is replaced by another natural variant with advantageous characteristics (*middle*). While the intrinsic interactions within the transferred module should already be optimal (*thick dotted lines*), perturbation of interactions (*thin dotted lines*) with extrinsic components might affect the overall function of the process. In that case, these extrinsic components must also be exchanged or suitably modified by laboratory evolution (*right*) to accommodate the chimeric multiprotein complex, perhaps also involving adaptive sequence variation (indicated by altered shading) of contact sites in the complex itself. Note that this approach is not likely to succeed for PSII (see main text) but might be feasible for other instances of FMAs

Although FMAs, by definition, are largely refractory to natural evolution, screening of existing biodiversity might nevertheless provide variants that mitigate their negative effects. For PSII, and in particular the D1 protein, the isolation of the non-model green alga *Chlorella ohadii* (from desert soil crusts) and its physiological characterization may lead to a breakthrough in enhancing the light resistance of plant PSII [63–66]. Even under extremely high irradiation intensities, growth of *C. ohadii* remains unimpaired and its photosynthetic O_2_ evolution actually increases at high light levels [63]. In contrast to other photosynthetic eukaryotes, very little of its D1 is degraded under such conditions, indicating that (i) PSII in *C. ohadii* might generate fewer ROS than do canonical PSII complexes [63] or (ii) PSII is less accessible to or better protected against ROS. Because green algae and flowering plants employ similar PSII complexes, with light-harvesting proteins as antennas (Fig. 1b), transfer of the entire *C. ohadii* PSII core module into a flowering plant should be more straightforward than that between cyanobacteria and flowering plants (see above), assuming that efficient expression of all components can be achieved, and biogenesis of the complex does not require (too many) algal-specific auxiliary proteins. However, although the molecular mechanisms of the high light tolerance of *C. ohadii* are still elusive [63], it appears to be unlikely that a modified PSII core is responsible for the enhanced light tolerance, with a combination of special antenna complexes and auxiliary factors providing a more plausible explanation. Moreover, the specialized ecological niche occupied by *C. ohadii* also raises the possibility of an as-yet unknown trade-off connected with the alga’s high light resistance, although enhanced growth of *C. ohadii* is observed even at low light intensities [65], which argues against the explanation that solely special antenna complexes (in terms of a reduced size) are responsible for the high light tolerance of this organism. But even if transfer of the PSII core of *C. ohadii* is not a promising approach to enhance light stress resistance, the question arises whether it is practicable to exchange entire PSII cores between green algae and flowering plants, thereby extending the approach of Gimpel et al. [50] to the next level of complexity. In fact, dysfunctional interactions between the transferred module and extrinsic components might affect the overall function of the process (Fig. 4). The latter can in principle be rectified by laboratory evolution (although this is not yet practicable in flowering plants) or by replacing the extrinsic components. A third possibility would be to streamline the plant PSII complex with a green algal core in a microbial test system that is amenable to genetic manipulation and laboratory evolution.

## Exploiting non-natural sequence variations to enhance PSII functions

While the exact number of natural protein sequences is unknown, it clearly represents a minuscule fraction of all possible proteins. Indeed, for a protein containing 100 amino acids, 20^100^ possible sequences exist, enough to fill a volume larger than that of Avogadro’s number of universes [67]. Certainly, only a small fraction of all possible proteins is compatible with biological systems, because many sequences would simply result in intrinsically disordered proteins. Of the compatible fraction, only a very tiny part has been exposed to evolutionary pressures on Earth, leaving a vast number of non-natural proteins capable of replacing existing natural proteins or mediating entirely novel functions. This concept is not only plausible; it has already been shown to be practicable [51]. Libraries of non-natural proteins with the potential to sustain the growth of living cells can be constructed in a systematic and knowledge-based manner, and non-natural proteins that perform specific functions have been identified by phenotypically complementing mutations in natural proteins [51]. This approach has resulted in the identification of non-natural proteins that rescued deletion mutants lacking natural proteins with various specific activities, including phosphoserine phosphatase, citrate synthase, threonine deaminase, and enterobactin esterase [68]. Moreover, established engineering parameters were used to generate simple non-natural four-α-helix bundles and build basic oxidoreductase activities into these scaffolds to create completely artificial redox proteins (“maquettes”) that can plug into natural biochemical pathways and are functionalized in vivo [52, 53, 69].

**Fig. 5.**
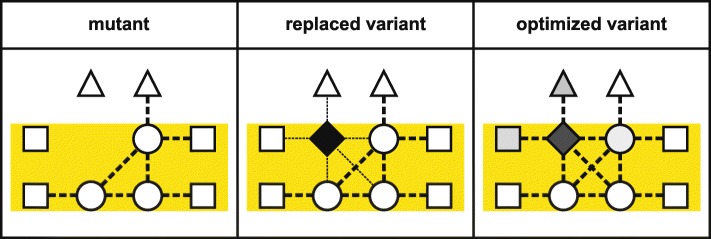
Resolving the consequences of FMAs using non-natural components. A similar workflow as for replacing the original variant by another natural variant (Fig. 4) is employed, except that, in the first step, the natural protein (*left*) is replaced by a functional non-natural protein from a library specifically designed for this purpose (*middle*). This can be achieved via homologous recombination (in the case of cyanobacterial or plastid transformation) or by complementation of a mutant that is grown heterotrophically (shown here). The function of the protein can be enhanced by laboratory evolution (*right*), optimizing interactions (from *thin dotted lines* to *thick dotted lines*) and involving adaptive sequence variation (indicated by altered shading) of the introduced non-natural protein and its binding partners

The central question in the context of PSII is whether a protein with the same tertiary structure and the same complex interaction pattern and cofactor-binding sites as a natural PSII core protein might be capable of substituting for that PSII protein even though their primary sequences might be entirely different. Indeed, analogously to the first test cases for non-natural proteins in *E. coli* described above, such an approach might employ libraries of non-natural proteins expressly designed to have features similar to those of the target to be replaced, e.g., the same number of transmembrane domains and the same cofactor-binding sites. Rescuing a mutant that lacks a specific natural protein with the aid of a suitable non-natural protein could then be accomplished by a complementation-based strategy, provided that the recipient can also be propagated heterotrophically (as in cyanobacterial and algal model systems). Alternatively, the heterotrophic step can be avoided if the natural protein is replaced by the non-natural one via homologous recombination, which is feasible in cyanobacteria and the chloroplasts of some eukaryotes. The resulting lines might then contain a non-natural protein that has replaced a natural co-adapted protein whose evolution had been constrained by a FMA (Fig. 5). The next step would be to optimize the introduced non-natural proteins by laboratory evolution, either by optimizing each protein before the next one is introduced or by introducing and optimizing them as a batch. In the former case, one might create a system comprised of components that display interactions and features similar to those of the original, but differ markedly from them in their primary sequences, whereas in the latter case interactions and features might differ substantially from the original. This concept is intriguing, but will be very difficult to implement. First, it requires detailed knowledge of the structure–function relationships of the proteins concerned. Even for the best-studied protein complexes—like the photosystems, RuBisCO, or ribosomes—our current understanding might not be sufficient. Secondly, a non-natural protein or maquette with similar structure and interactions to its natural “predecessor” but completely different in sequence might be refractory to the natural assembly process, making novel assembly factors necessary. Thirdly, as laboratory evolution is feasible only in microbial systems, if specific aspects of plant photosynthesis need to be re-designed, the corresponding components must first be transferred into a suitable microbial host. Furthermore, if a new system consisting of non-natural proteins displays the same intrinsic interactions, it might also represent a soon-to-be FMA. Finally, replacing the original proteins with non-natural proteins or maquettes with similar functions will not enhance the process if the basic structure of the process is inherently suboptimal, for instance if any heterodimerically organized reaction center of PSII inevitably leads to D1-based photoinhibition [70]. In the latter case, its re-design with non-natural proteins with similar characteristics will also be in vain, and the only solution is to redesign the process from scratch as outlined below.

## Redesign from (almost) scratch to enhance PSII functions

**Fig. 6.**
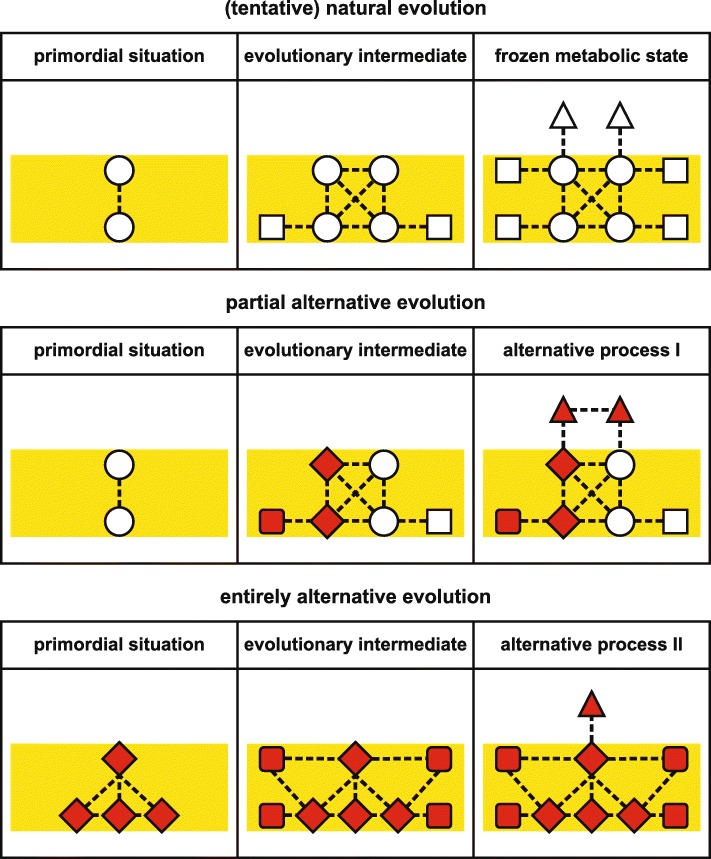
Comprehensive redesign of the process that is associated with the FMA. A primordial state of the process might be reconstructed by, for instance, comparing anoxygenic bacterial and oxygenic photosynthesis (*upper panels*). From this primordial process, an alternative version of the process to be replaced can be constructed in a step-wise manner, employing genetic engineering, as well as in vitro approaches using libraries of suitable compounds (*middle panels*). If a suitable primordial process cannot be reconstructed or is already flawed, the process might be re-designed from scratch, requiring deep knowledge of the original process (*bottom panels*)

How can the deleterious consequences of a FMA be redressed if the process itself is already intrinsically flawed? Or, with respect to PSII, what if all designs of water-splitting PSII found in extant organisms share the same inevitable flaw of photosensitivity and D1 damage? In fact, it can be argued that the energetics of the reaction mediated by PSII necessitates PSII being susceptible to ROS generation. In this scenario, re-designing PSII such that it circumvents ROS production is impossible, although it might well be engineered so that less ROS are produced, ROS are better scavenged, or that central parts of the complex are less accessible to ROS. Another thought is that a certain level of photosensitivity of PSII has been positively selected for during evolution because this serves to protect PSI against photoinhibitory damage, which is considered to be more dangerous than PSII photodamage [71–74]. In this case, high light tolerance of both PSII and PSI has to be enhanced to have a positive impact on the resistance of the photosynthetic light reactions towards high levels of light, and only two enhancement strategies remain open: (1) re-design of the process, starting from a primordial version that does not exhibit the relevant defect(s), or (2) complete replacement with a synthetic process designed from scratch (Fig. 6). With regard to the former possibility, all natural light-dependent and water-splitting systems in cyanobacteria, algae, and plants are related to each other, but anoxygenic bacterial relatives of PSII have a less complex structure and might serve as models of primordial versions of PSII [70, 75–78]. Starting from these primitive versions of PSII, one needs to recapitulate the evolution from non-oxygenic, bacterial, single-photosystem situations to a cyanobacterial, oxygenic, two-photosystem solution that operates optimally in the presence of oxygen and high light. This option presents various formidable technical and conceptual obstacles, and will require a more detailed understanding of the mechanisms and evolution of photosynthesis than has yet been attained. The second possibility—inventing a synthetic system that is devoid of the flaws of the natural one—is even more challenging. Although it is clear that water splitting capacity will always be associated with the potential to produce ROS and that it is unlikely that a new type of in vivo photosynthesis can be constructed from scratch in the near future, the associated opportunities are fascinating, as one could also combine chlorophylls and bacteriochlorophylls in the same organism, or even incorporate novel pigments that absorb more of the light spectrum than conventional ones can utilize.

## Harnessing synthetic biology to resolve frozen metabolic accidents

Due to their immense significance for the biosphere in terms of nitrogen and carbon fixation, efforts to enhance the processes associated with FMAs are of the utmost interest. Elegant manipulations of the genetic code have demonstrated that it is feasible to get around the frozen *genetic* accident, but frozen *metabolic* accidents involve another level of complexity, owing to the intricate biophysical and structural interdependencies implicated in the frozen state, which extend to auxiliary components. To resolve the roadblocks caused by FMAs, a comprehensive knowledge of the process to be modified is vital, and elegant genetic engineering strategies have to be designed. The most promising and technically feasible approaches of resolving FMAs currently are to exploit the few evolutionary opportunities that they have left open by screening extant biological diversity for possible remedies (e.g., the high-light resistant PSII from *C. ohadii*) and applying laboratory evolution in cases where the process can be transferred to a suitable host (as in the case of nitrogenase and the Calvin cycle in *E. coli*). A much more challenging approach will be to test large sets of non-natural proteins or maquettes for their ability to overcome some of the limitations imposed by FMAs, and this will be only possible in suitable microbial systems that are accessible to efficient genetic engineering and laboratory evolution. The ultimate challenge will be to redesign from scratch a process that has been trapped in an evolutionary blind alley and is in principle suboptimally adapted to current conditions, at least in terrestrial environments. However, replacing this process with a completely different one amounts to discovering an evolutionary path that was never realized in nature, because the appropriate combination of starting conditions and selective forces never materialized. In this context it remains to be shown whether PSII truly qualifies as FMA because its sensitivity to light stress due to ROS production might have evolved regardless of the conditions (aerobic or pre-aerobic) and might therefore be inevitable, although it is clear that at least some organisms apparently have developed mechanisms that can largely overcome this limitation (the *C. ohadii* case). Moreover, the photosensitivity of PSII also protects PSI against photoinhibition, extending the need for re-design of both photosystems. Whichever explanation(s) for the inherent light sensitivity of PSII will finally turn out to be correct, for enhancing the function of a process assumed to be a FMA one might ideally start from a primordial process that is sufficiently flexible to allow it to be re-designed by employing non-natural proteins and laboratory evolution. For such experiments, *E. coli* is the ideal workhorse, and given that fixation of nitrogen [44] and carbon [30] and the biosynthesis of chlorophyll [79] and carotenoids [80–82] have already been reconstituted in this host, the light reactions of photosynthesis are an especially attractive target of this approach. Thawing out FMAs will, however, require synthetic biology and laboratory evolution, but also further knowledge of the reactions themselves.
